# Extension of Genetic Marker List Using Unnatural Amino Acid System: An Efficient Genomic Modification Strategy in *Escherichia coli*

**DOI:** 10.3389/fbioe.2020.00145

**Published:** 2020-04-28

**Authors:** Xinyi Xu, Huichang Zhong, Weifeng Liu, Yong Tao

**Affiliations:** ^1^Engineering Research Center of Molecular and & Neuroimaging, Ministry of Education, School of Life Sciences and Technology, Xidian University, Xi’an, China; ^2^Chinese Academy of Sciences Key Laboratory of Microbial Physiological and Metabolic Engineering, Institute of Microbiology, Chinese Academy of Sciences, Beijing, China; ^3^Xiamen Huison Biotech Co., Ltd., Xiamen, China

**Keywords:** unnatural amino acids, Cre-mediated recombination system, antibiotic resistance gene, neurosporene, *Escherichia coli*

## Abstract

Genetic manipulations including chromosome engineering are essential techniques used to restructure cell metabolism. Lambda/Red (λ/Red)-mediated recombination is the most commonly applied approach for chromosomal modulation in *Escherichia coli*. However, the efficiency of this method is significantly hampered by the laborious removal of the selectable markers. To overcome the problem, the integration helper plasmid was constructed, pSBC1a-CtR, which contains Red recombinase, Cre recombinase, and exogenous orthogonal aminoacyl-transfer RNA (tRNA) synthetase/tRNA pairs, allows an unnatural amino acid (UAA) to be genetically encoded at the defined site of the antibiotic resistance gene-encoded protein. When UAAs are not in the culture medium, there was no expression in the antibiotic resistance gene-encoded protein. Accordingly, the next procedure of antibiotic gene excising is not needed. To verify this method, *pox*B gene was knocked out successfully. Furthermore, sequential deletion of three target genes (*gal*R, *pts*G, and *pgi*) was able to generate neurosporene-producing strain marked by high growth rate. Thus, the site-specific incorporation UAA mutagenesis system were used to control and expand the use of conditional selectable marker, and the technique is used to facilitate a rapid continuous genome editing in *Escherichia coli*.

## Introduction

Synthetic biology methods have greatly facilitated the optimization of processes in metabolic engineering. New promoters, ribosome binding sites, and gene circuits were designed using synthetic technologies and assembled into metabolic pathways to improve the metabolic flux and gene regulation efficiency. By these ways, the production of medicinal chemicals such as artemisinin (Chang et al., 2007) and taxol (Li et al., 2019) has been successfully optimized. Continuous genomic modification is an important tool for the optimization of processes in metabolic engineering. The λ/Red-based system was developed to mediate chromosomal homologous recombination, and resistance gene markers were used for genetic selection (Wang et al., 2016). The three plasmids of pKD4, pKD46, and pCP20 are widely used in λ/Red-mediated recombination. The pKD46 vectors include three genes: γ, β, and *exo*, whose products make chromosomal recombination. The plasmid pKD4 (or pKD3, pKD13) was template vector. The PCR products were generated using pairs of primers that included homology extension sequences of targeted gene and priming sequence of plasmid pKD4 (or pKD3, pKD13). The plasmid pCP20 has a temperature-sensitive replicon, antibiotic resistance marker gene, and flipase recombination enzyme (FLP) gene. FLP acts on the directly repeated FLP recognition target (FRT) sites flanking the resistance gene and makes FRT-flanked resistance gene to be eliminated. After one round of homologous recombination experiment, the vector pCP20 was electroporated into the host cells to eliminate the resistance gene, and the cells get ready to go to the next round of experiment when the pCP20 vector is lost from the cell under 42°C (Datsenko and Wanner, 2000). Although the efficiency of elimination of the antibiotic resistance gene is high (more than 80% cells lost the antibiotic resistance gene in genome), more than 80–100 nt scar, which included an FRT site, would be left behind the disrupted genes. After more than five times of chromosomal recombination experiments, the scars as mentioned above present in the host chromosome can influence expression of surrounding genes and caused the failure to achieve continuous knockout or integration experiments (Madyagol et al., 2011). To avoid this shortage, two rounds of recombination were designed and carried out by Zhang et al. (2007). In the first recombination, some of the target genes were replaced by a DNA cassette containing a chloramphenicol resistance gene (Cm^R^) and a left sucrase gene (*sacB*). In the second recombination, the Cm^R^*–sacB* cassette was eliminated by the selection for resistance to sucrose. However, this method also has shortcomings. The efficiency of the first homologous recombination was low, which may be due to the fact that *sacB* gene also had certain toxicity to *Escherichia coli* (*E. coli*) in the absence of sucrose. During the first homologous recombination, most of them were inactivated mutations of *sacB*, which led to a false positive when adding sucrose to screen the recombinant cells to eliminate *sacB*. Beyond that, the experimental procedure was laborious and time consuming, as the vector was electroporated into the host cells and eliminated from the cell under 42°C. Song and Lee developed an integration plasmid that harbors both λ/Red recombinase and *cre* gene under the control of two independent promoters (Song and Lee, 2013). However, in this system, the template plasmid does not contain multiple cloning sites, and the conventional p15A replication initiation site is used, which is not conducive to gene integration operations. So this method has not improved the positive rate. Researchers wanted to develop a plasmid strategy that mediates rapid one-step inactivation of genes. CRISPR-Cas9 system is an efficient genomic editing technique that is be developed (Cho et al., 2017). Unfortunately, due to the lack of homologous repair system in *E. coli*, the application of CRISPR-Cas9 is hampered and can be used in combination with the λ/Red recombination system. During these procedures, although selectable markers are not used, longer homologous arms and higher integration efficiency are required to ensure genomic integration. Furthermore, to recognize plasmids during each round of genomic modification, the protospacer adjacent motif site should be redesigned, which complicates the process.

The genetic encoding of unnatural amino acids (UAAs) are among the most extensively explored artificial systems. Incorporating UAAs into protein using evolved orthogonal aminoacyl-transfer RNA (tRNA) synthetase/tRNA pairs could confer the protein novel chemical and physical properties. Currently, UAAs have been wildly used in diverse bio-research fields (Young and Schultz, 2018), including exploration of protein structures, protein posttranslational modifications, and photo-cross-linking, and other specific therapeutic applications. For example, the abiotic cofactor nicotinamide flucytosine dinucleotide can be used to improve the efficiency of biological redox reactions (Ji et al., 2011). Thus, the unnatural biological molecules could provide an independent orthogonal system that does not interfere with the complex natural network in the living cell. This advantage indicates a potential application of the unnatural compounds in metabolic engineering.

In this study, we propose that UAAs can serve as a switch to turn on the specifically designed cell conditions in metabolic engineering. We generated a conditional selectable marker (CSM) by introducing the amber codon UAG into the sequence of selectable marker genes. The resistance function of these genes is eliminated in normal condition, whereas it is regained in a 3-iodo-L-tyrosine plate when corresponding orthogonal aminoacyl-tRNA synthetase/tRNA UAG pairs are introduced. This CSM system expands the power of genomic modification via reuse of the selectable marker. As CSM can be obtained for different resistance genes, the selectable marker list is significantly expanded, and continuous genomic modification can be carried out in multirounds without curing of resistance marker genes. Furthermore, all resistance marker genes can be removed in one go using Cre/*lox* recombination. In addition, different incompatible variants of *lox* site, including flank variant *lox*66/71 and spacing variants *lox*511, *lox*2272, m3, and m5, can provide stable curing of selectable markers at each genomic modification site. Considering that CSM can be further expanded by other UAA systems, this work will provide a rapid genomic modification strategy that can be efficiently used in metabolic engineering.

**GRAPHICAL ABSTRACT:**
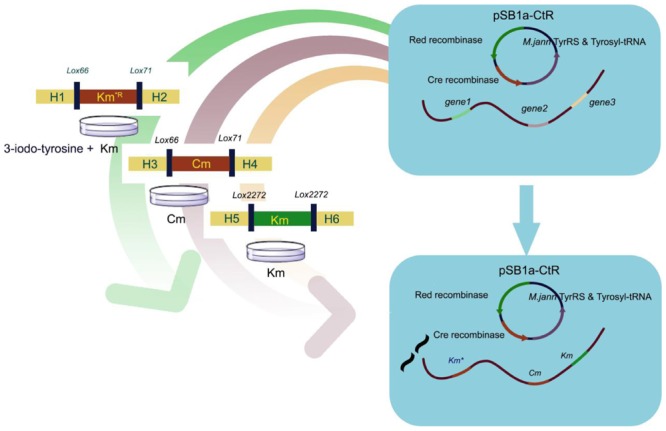
Simultaneous triple gene deletion experiments. In the first deletion procedure, the selective marker Km^R^ gene 160th site codon was mutated to the amber stop codon. Cells were spread onto agar with UAA to select the Km^R^ transformants. After confirmation by PCR, the cells were processed to knockout genes in experiments with different antibiotic-resistant genes and *lox*P site without iterative plasmid transformation or curing steps.

## Materials and Methods

### Bacterial Strains and Culture Conditions

Strains and plasmids used in this study are listed in Table 1. *E. coli* BW25113 was used as a host strain in gene knockout experiments, whereas the *E. coli* strain DH5α (TransGen Biotech Co., China) was used in all cloning experiments. For the construction of plasmids and strains, *E. coli* cells were grown in Luria–Bertani (LB) medium or on medium plate with 1.5% agar at appropriate temperature. Bioconversion medium was prepared by adding 4% (*w*/*v*) glucose to the Minimum Essential medium. The appropriate antibiotics were added in the following concentrations: 50 or 100 μg/ml of kanamycin, 100 μg/ml of ampicillin, and 25 μg/ml chloramphenicol. A total of 1 mM isopropyl-β-D-thiogalactoside (IPTG, Sigma-Aldrich) and 0.2% L-arabinose (Sigma-Aldrich) were used for induction. The transformed bacterial cells were grown at 30°C in LB medium plate supplemented with 1 mM 3-iodo-L-tyrosine (Sigma-Aldrich). All flask cultures were grown in ZYM-5052 autoinducing medium supplemented with glucose and with appropriate antibiotics (Studier, 2005). For the cultivation of an engineered *E. coli* strain for neurosporene production, a preculture was prepared by inoculating a single colony, which was stored at −80°C until use, in a 25-ml test tube containing 5 ml LB medium and cultivated at 37°C and 220 rpm in a shaking incubator after thawing. Cells (3 ml) that had grown for 12 h were transferred to a 300-ml baffled flask containing 100 ml ZYM-5052 autoinducing medium and were incubated for further 12 h. Following this, 10 ml 40% (wt/vol) of glucose was added to the baffled flask and incubated for 24 h.

**TABLE 1:** Bacterial strains and plasmids used in this study.

**Strain or plasmid**	**characteristic(s)**	**References or sources**
*E. coli* BW25113	*lacI*^q^ *rrnB*_T__14_ Δ*lacZ*_WJ__16_ *hsdR514*Δ*araBAD_AH__33_*Δ*rhaBAD_LD__78_*	Lab stock (Datsenko and Wanner, 2000)
*E. coli* DH5α	*F-supE44*λ*-*Δ*(lacZYA-argF) U169* (Φ*80lacZ*Δ*M15) hsdR17 recA endA1 gyrA96 thi-1 relA1 phoA*	TransGen Biotech
*E. coli* PY0012	*E. coli* BW25113, Δ*poxB:p119-ispDF-Km*	This study
*E. coli* PY2006	*E. coli* BW25113, Δ*galR*,Δ*ptsG:p119-glk*,Δ*pgi*	This study
pKD46	Ap^R^; λ-Red recombinase under araBAD promoter; ts origin	Lab stock (Datsenko and Wanner, 2000)
pC1	Cm^R^; C1 promoter; pSC101 origin	Lab stock, Unpublished
pYC1c	Cm^R^; Tac promoter; p15A origin	Lab stock
pSB1s	Str^R^; araBAD promoter; pSC101 origin	Lab stock
pBK	Km^R^; TyrRS under GlnRS promoter; ColE1 origin	Lab stock (Wang et al., 2006)
pSLM	*Lox*66-Km^R^-*Lox*71; MCS; pSC101G93R origin	Lab stock (Wang et al., 2015)
pS93S	Str^R^; p119 promoter (BBaJ23119 in the registry for standard biological parts; www.partsregistry.org); pSC101 origin	Lab stock
pYC1c-Cre	Str^R^; Cre recombinase under Tac promoter; p15A origin	This study
pSBC1a-Cre	Ap^R^; λ-Red recombinase under araBAD promoter; Cre recombinase under Tac promoter; ts origin	This study
pC1RT	Cm^R^; TyrRS under C1 promoter; three copied *Tyrosyl-tRNA* under lpp promoter; pSC101 origin	This study
pSBC1a-CtR	Ap^R^; λ-Red recombinase under araBAD promoter; Cre recombinase under Tac promoter; TyrRS under C1 promoter; three copy Tyrosyl-tRNA under lpp promoter; ts origin	This study
pSLKM	*Lox*66-Km^R^-*Lox*71; MCS; pSC101G93R origin; Kan gene site 160th mutate to UAG	This study
pSLC	*Lox*66-Cm^R^-*Lox*71; MCS; pSC101G93R origin	This study
pSLK2272	*Lox*2272-Km^R^-*Lox*2272; MCS; pSC101G93R origin	This study

*Ap^*R*^, ampicillin resistance gene; Cm^*R*^, chloramphenicol resistance gene; Km^*R*^, kanamycin resistance gene; TransGen Biotech, TransGen Biotech Co., LTD, China.*

### Selecting Variants and Increasing tRNA Levels to Increasing the Unnatural Amino Acid Incorporation Efficiency

The candidate variants including the six different mutation sites within the active center of the sequence of kanamycin resistance gene (aminoglycoside kinase APH 3′IIa, Km^R^) were designed and obtained. The mutations were obtained using the PCR method. With the addition of 1 mM of 3-iodo-L-tyrosine, the growth state of the strains that harbor plasmids containing Km^R^ mutants were detected by means of kanamycin plate coating. Accordingly, we improved the expression level of tyrosyl-tRNA synthetase (TyrRS) using promoter replacement by strong constitutive PC1 promoter and placed three copies of tRNA^*tyr*^ (CUA) to get pC1TR.

### Constructing the pSBC1a-CtR, pSLKM, pSLC, and pSLK2272 Vectors

Gene cloning procedures, including PCR amplification, restriction, and ligation, were carried out according to the established protocols. All oligonucleotides are listed in the supporting information (Supplementary Table S1). *E. coli* cells were transformed by the electrotransformation method. The Gibson method was used for assembling multiple overlapping DNA fragments into a plasmid in single step without any restriction enzyme sites.

To improve the efficiency of continuously conducted gene modification, we optimized the plasmids used in the developed UAA conditional selectable marker (UCM) system. First, an integrated plasmid pKD was obtained using pKD46 as template. The TryRS gene and triple tRNAs were inserted into pKD46. The plasmid pC1RT that contains artificial aminoacyl-tRNA synthetase/tRNA pair was constructed. The pYC1c plasmids were double digested by *Kpn*I and *Eco*RI and then ligated to the *cre* gene, which was synthesized using the vector pJL519 (Patel et al., 2010) and was amplified by PCR (Cre-*kpn*I-F and Cre-*Eco*RI-R). The final vector was named pYC1c-Cre. The vector pC1 was digested with *Nco*I and *Xho*I and assembled with the TyrRS gene, which were amplified from pBK-Ityr plasmid by PCR (Wang et al., 2006). The TyrRS gene and three copies of *tyrosyl-tRNA* genes were cloned into a module plasmid pC1RT using the Gibson assembly method (Davidson et al., 2002). Vector pKD46 was digested to linear segment, and *cre* genes under P_Tac_ promoter were amplified from the plasmid pYC1C-Cre. The TyrRS gene under C1 promoter and three copies of tRNA under lpp promoter were amplified from plasmid pC1RT by PCR. The three aforementioned DNA segments were integrated using pSBC1a-CtR plasmid by the Gibson method.

Three types of template plasmids containing antibiotic resistance genes flanked by the mutated *lox*P sites were used in PCR amplification. The first plasmid, pSLKM, contains the *lox*LE-Km-gene-*lox*RE region constructed in our previous study (Wang et al., 2015) and contains mutation Km^R^. The second plasmid, pSLC, had the same backbone as pSLM, but contained Cm^R^, a different antibiotic gene. To construct the plasmid pSLC, the PCR products containing Cm^R^ gene that flanked *Not*I and *Nhe*I restriction sites from pSC1c (our lab stocked) were ligated into vector plasmid pSLM digested by restriction endonuclease *Not*I and *Nhe*I. The third plasmid, pSLK2272, was the same as pSLM, but contained *lox*2272 sites flanking the Km^R^ gene. To construct pSLK2272, the Km^R^ gene was amplified from the plasmid pKD4 using the primers lox2272Km-F and lox2272Km-R. The pSLM plasmids were digested into linear sequence by restriction endonuclease *Not*I and *Nhe*I. The PCR products included the *lox*2272-Km^R^-*lox*2272 gene and were ligated into linear pSLM fragments using the Gibson method. The above DNA sequence was confirmed by genetic sequencing.

### PCR Amplification and Electroporation of Knockout Fragments

To validate our method, the pSLKM plasmid was used as a template vector for the amplification of the linear gene and knockout of DNA fragments. This pSLKM vector was digested into the linear gene by restriction endonucleases and ligated to recombination gene. Each linear knockout fragment was amplified by overlapping two PCR procedures. The first PCR was performed using the primers F1 and R1 for each gene, which would be knocked out. F1 sequence has 70-bp homologous arm sequences of the target gene. R2 has internal homologous sequence of the according antibiotic resistance gene. The second PCR was performed using primers (F2 and R1). F2 sequence has internal homologous sequence of the according antibiotic resistance gene. R1 has 70-bp homologous arm sequences of the target gene. The two PCR products that contained 20-bp overlapping gene sequences were used as templates to perform the final PCR using the primers (F1 and R1) to create full-length PCR product. The PCR product was named poxB-PCR, which included 70-bp homologous arm sequences of *poxB*, *lox*LE/RE site, and Km^R^ mutation gene with an amber codon UAG substituted for the 160th site.

For simultaneous three-gene knockout experiments, the pSLKM, pSLC, and pSLK2272 plasmids were used as template vectors to amplify the linear PCR product. DNA amplification was the same as the two overlapping PCR procedures described above. All the primers used in this study are listed in Supplementary Table S1. The first PCR product was named galR-PCR and had 50-bp homologous arm sequences of *GalR, lox*LE/RE site, and Km^R^ with an amber codon substituted for 160th site in the mutation gene. The second PCR product amplified from the pLSC-glk vector, in which the *glk* gene with p119 promoter were inserted to pLSC by the Gibson method, was named ptsG-PCR and had 50-bp homologous arm sequences complementary to the downstream and upstream regions of the *ptsG* gene, respectively (the primers were glk-F1 and glk-R1). The third PCR product was named pgi-PCR and was constructed using the pSLK2272 vector and the primers pgi-F1 and pgi-R1 with 50-bp homologous arm sequences complementary to the downstream and upstream regions of the *pgi* gene, respectively. PCR products were gel purified, digested with *Dpn*I, repurified, and suspended in an elution buffer (0.5 mM Tris, pH 8.0). Transformants carrying pSBC1a-CtR plasmid were grown in LB medium with ampicillin and L-arabinose and harvested at an OD_600 nm_ of about∼0.5 by centrifugation at 1122 × *g* and 4°C. Cell pellets were washed twice with 10% (*w*/*v*) glycerol and suspended in 100 μl of the same glycerol solution.

### Single, Sequential Multiple Gene Deletions

For the gene knockout experiment, the procedure was as follows. First, approximately 1–2 μg of PCR products was added to the 200 μl *E. coli* BW25113 competent cells containing the pSBC1a-CtR plasmid. Electroporation was then performed using Gene pulser II (Bio-Rad) and 2-mm electroporation gap cuvettes (Bio-Rad). The electroporated cells expressing λ-Red recombinase were transferred with the linear gene knockout DNA fragment to trigger a recombination event. Transferred cells were incubated at 30°C, and the positive colonies were selected on LB agar plates containing 1 mM 3-iodo-tyrosine and 50 μg/ml kanamycin. Positive recombinants were verified by colony PCR using two confirming primers. The primer F1 is a copy of the sequence to the outside left of the target gene. The primer R1 is a copy of the sequence to the outside right of the target gene. The primer Km-TAA-F is a copy of the reverse sequence originated from UAA codon of the Km^R^ gene. The primer Cm-TAA-F is a copy of the reverse sequence originated from UAA codon of the Cm^R^ gene. The confirmed colonies were streaked and grown on LB agar plates containing 1 mM IPTG and ampicillin to excise the Km^R^ and Cm^R^ genes. Control colonies were always tested side by side. All the primers used in this study are listed in Supplementary Table S1.

To test our method, simultaneous triple gene deletion experiments were conducted using three selected target genes *galR*, *ptsG*, and *pgi*. In some experiments, *glk* gene with p119 promoter was used instead of gene *ptsG*. The selection and transfection procedures were the same as described above. Cells that contained the galR-PCR product were incubated for 1 h at 30°C. The cells were then spread for 30 min on agar with 3-iodo-Tyr to select Km^R^ and ampicillin resistance gene (Ap^R^) transformants at 30°C. Recombinants were confirmed by colony-testing PCR with primers as noted above using the forward primer Km-TAA-F and the reverse primers *galR*-F. The confirmed single colony with knocked out *galR* was selected. After the first step, the ptsG-PCR products were added to the 200-μl confirmed cells harboring the pSBC1a-CtR plasmid. The cells had been incubated at 30°C for 1 h, and then half was spread onto agar to select Cm^R^ and Ap^R^ transformants at 30°C. Recombinants were confirmed by colony-testing PCR using the forward Cm-TAA-F primer and the reverse PTSG-RC primer. The final confirmed colonies were spread and grown on LB agar plates containing kanamycin and ampicillin. The fragment of Km^R^ gene was amplified from plasmids pSLK2272 using primers pgi-F1 and pgi-R1, then was electrotransfected into cells harboring pSBC1a-CtR. The transformants were spread on plate containing kanamycin and ampicillin to screen for positive colonies. In the final step, 1 mM IPTG was added to the culture medium, and the cells were induced by the *cre* recombinase expression to excise the three resistance genes simultaneously. After all sequential gene deletion experiments had been completed, pSBC1a-CtR was eliminated at 42°C.

### The Neurosporene Synthesis Procedures

To confirm the efficiency of this method for the sequential deletion of multiple genes, three genes were sequentially deleted to develop a neurosporene-producing *E. coli* strain as a proof-of-concept example. This strain was developed by the deletion of the *galR*, *ptsG*, and *pgi* gene; meanwhile, *glk* genes were increased in *E. coli*. During the cultivation, cell turbidity was monitored spectrophotometrically at 600 nm with a UV-1800 spectrophotometer (SHIMADZU, Japan). Neurosporene was extracted from cell pellets with acetone under agitation conditions, and its concentration was determined using absorption spectrum at 470 nm using a UV–visible (UV–vis) spectrophotometer. Neurosporene content was calculated by normalization of neurosporene production to dry cell weight (Farasat et al., 2014).

## Results

### Development of Conditional Selectable Marker Using UAA

CSM was created by insertion of amber stop codon UAG to replace the sense codons within the sequence of Km^R^. We mutated the six genes of different amino acid sites to UAG within the active center of Km^R^. We compared the impact of different mutations on the loss of kanamycin-resistance phenotype. Strains harboring pC1RT vector and either of the pSLM plasmids that contained the mutated Km^R^ genes did not grow on kanamycin plates (Supplementary Figure S1, left). When 1 mM of 3-iodo-L-tyrosine was added, all of these strains grew (Supplementary Figure S1, right). We selected the mutation with the Km^R^ gene of GLU160 to UAG.

The evolution from *Methanococcus jannaschii* TyrRS that charges a modified *M. jannaschii* tRNA^*tyr*^(CUA) with 3-iodo-L-tyrosine was expressed under P*_C__1_* constitutive promoter site, whereas orthogonal tRNA gene was expressed under its native P*_Lpp_* promoter. Accordingly, we improved the expression level of placed three copies of tRNA^*tyr*^ (CUA). As expected, the BW25113 cells (contained three plasmids: pLSM with Km^R^ 160th site mutation, pC1, pKD46) grew well on the plates containing kanamycin, chloramphenicol, ampicillin, and 3-iodotyrosine (Supplementary Figure S2).

### Recombinant DNA Was Used for the Development of a Knockout System

**FIGURE 1:**
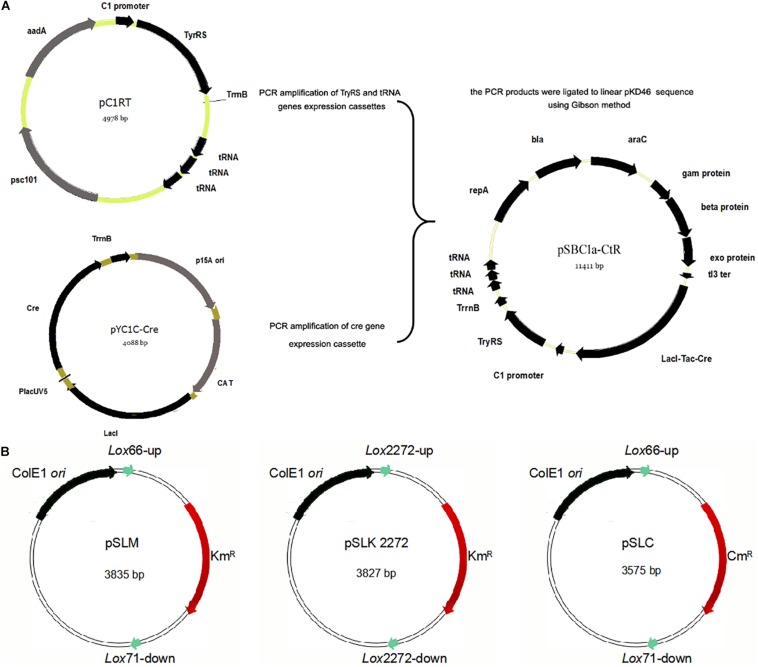
Construction of the integration helper pSBC1a-CtR and three template plasmids. **(A)** The IPTG-inducible Red recombinase gene under P_Tac_ promoter was amplified from pYC1c-Cre and the TyrRS gene by PCR. Three copies of transfer RNA (tRNA) were amplified from plasmid pC1RT by PCR. Vector for construction of the pSBC1a-CtR plasmid by overlap PCR method was used to create the three copies of Tyrosyl-tRNA PCR product. TyrRS gene PCR products and three copies of Tyrosyl-tRNA PCR products were constructed using the vector named pC1RT via Gibson method. pKD46 was digested to linear segment and ligated to the PCR products mentioned above by Gibson method. The final vector was named pSBC1a-CtR. **(B)** Map of template plasmids pSLKM, pSLC, and pSLK2272 is shown.

The knockout system mainly includes two helper plasmids. As one of the two helper plasmids, the integration helper plasmid pSBC1a-CtR was constructed and was composed of arabinose-inducible Red recombinase gene, Cre recombinase gene, and exogenous orthogonal aminoacyl-tRNA synthetase/tRNA genes. The cells containing the plasmid pSBC1a-CtR was able to express the active antibiotic resistance gene-encoded protein that was with an UAA at the defined site (Figure 1A and Supplementary Table S3). To eliminate the background expression of *cre* gene under the control of P_Tac_ promoter, all manipulations were performed on plates supplemented with 1% glucose.

To obtain a selectable marker-free genetic background for further rounds of modifications, it is necessary to remove the selectable markers used in each previous round. In the developed UCM system of genomic modification, we have introduced different *lox* variants including *lox*2272/*lox*2272 and *lox*66/71 that were flanked to the selectable marker genes. The plasmid pSLKM (or pSLC, pSLC2272) (Figure 1B) was used as the template vector. The template vector has two functional areas. First, the *Lox*66/*Lox*71 or *Lox*2272/*Lox*2272 sequences were the action sites of FLP. The FRT-flanked resistance genes were lost under action of FLP (Cre recombinase). Second, the multicloning sites (MCSs) were on one of the two sides of the resistance marker gene. The sequence of the MCS and FRT site sequence is shown in Supplementary Table S2. Subsequently, multiresistance markers were excised simultaneously when the Cre recombinase was expressed on an LB agar plate containing ampicillin and IPTG. The excision of four resistance markers was confirmed using colony-based PCR.

### Genome Modification Using UCM System

**FIGURE 2:**
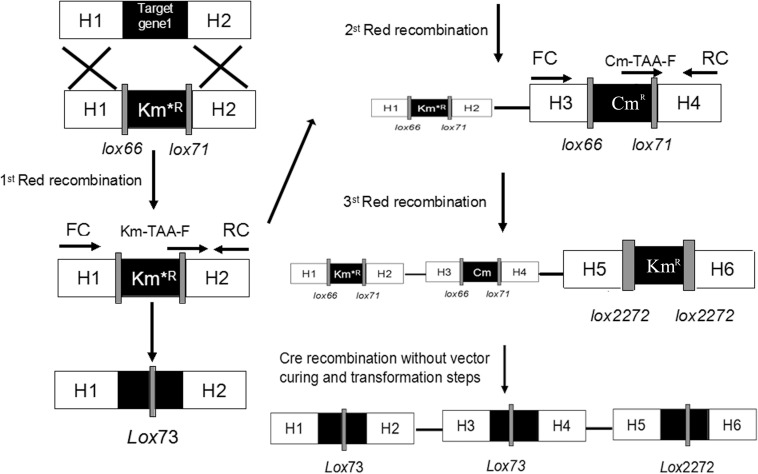
Schemes for gene knockout and excision of markers. Procedures are shown for the single-gene deletion **(left)** and simultaneous tripe-gene deletion **(right)** using the pSBC1a-CtR plasmids.

**FIGURE 3:**
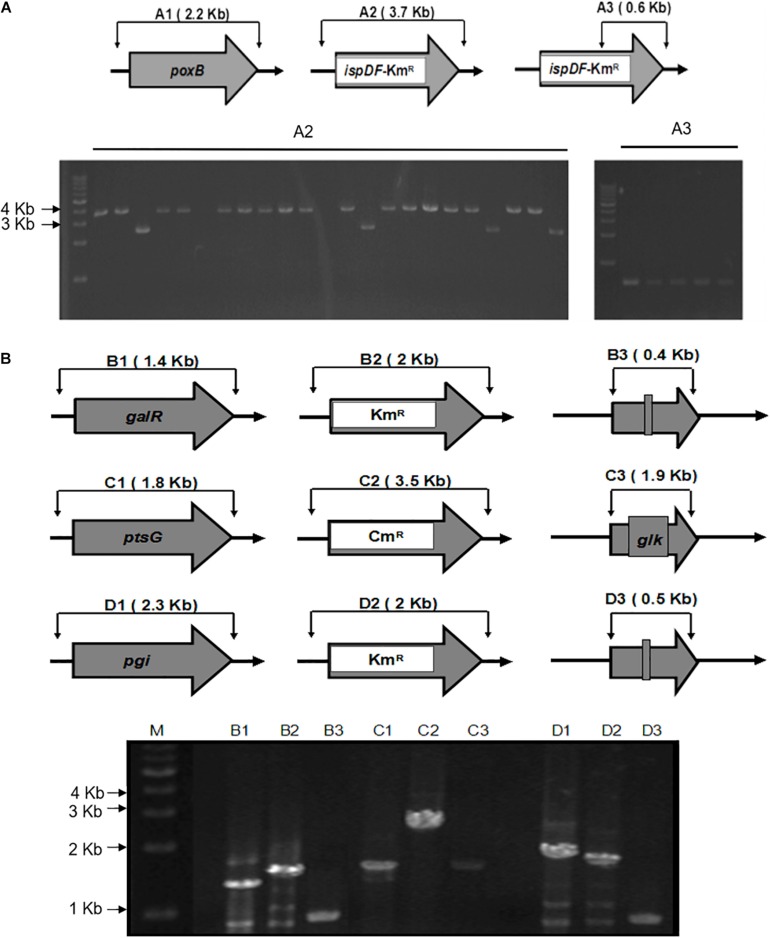
Schemes for gene knockout and excision of markers. **(A)** The gel image of PCR results is shown for the single knockout of the *poxB* genes and replacing with *ispDF* gene. A1, wild type; A2 and A3, mutants with a resistance marker and *ispDF*. A1 and A2 knockout mutants were confirmed using the FC and RC primers for each gene. A3 mutants were confirmed with resistance markers using the RC primers for each gene and the Km-TAA-F primer. **(B)** The gel image of the PCR-amplified chromosomal DNA fragments. The gel image of PCR results during the triple knockout of the *gal*R, *pts*G, and *pgi* genes is shown. B, *gal*R; C, *pts*G; D, *pgi*; 1, wild type; 2, mutant with a resistance marker; 3, knockout mutant; M, marker. Routine colony PCR was performed to check the recombination events. Wild-type and knockout mutants were confirmed using the FC and RC primers for each gene.

To illustrate the application of UCM system for the modification of chromosomal genes in *E. coli*, the mutant Km^R^ was used as selectable marker for the deletion of *poxB* gene. Replacing the *poxB gene* with the *ispDF* gene can result in redirection of central carbon flux and lead to various metabolic responses. The fragment of Km^R^ gene was amplified from plasmids using primers, then was electrotransfected into cells harboring pKD46 and pC1RT. The process of the recombination is presented in Figure 2 (left). The transformants were spread on the plate containing kanamycin and 1 mM 3-iodo-L-tyrosine to screen for the positive colonies. Altogether, 21 colonies were picked out, and gene expression was confirmed using PCR method with primers RC and Km-TAA-F, as shown in Figure 2 (left). All colonies were tested positively. The five colonies were chosen randomly and further verified by PCR and sequenced to prove the correct expression. The rate of selected positive clone was 83% (Figure 3A). This result indicates that the CSM can be used for chromosomal modification.

### Multiple Genomic Targets Were Modified Sequentially Using UCM System

As shown in Figure 2, the fragments that contain UAG mutational kanamycin gene flanked two homology regions at galR gene site were amplified for the first round of deletion. The plasmid pSLC was used as a template for PCR amplification. Targeted fragments were then transferred into the cells. The transformants were spread on the plate containing kanamycin and 1 mM 3-iodo-L-tyrosine to screen for positive colonies. Chosen positive strains were subjected to the next round of deletion. Then, the PCR product that replaced the ptsG with glk was transformed to the cells without removing the mutated Km^R^ and galR gene loci. The transformants were screened on chloramphenicol plates. The chosen positive strains were subjected to the next round of deletion. The obtained double-genes deletion strain that retains pSBC1a-CtR was subjected to further rounds of gene deletion. In the third round of experiments, deletion of *pgi* can result in the redirection of central carbon flux and lead to various metabolic responses. The fragment of Km^R^ gene was amplified from plasmids using primers, then was electrotransfected into cells containing pSBC1a-CtR from the last deletion experiment. The transformants were spread on the plate containing kanamycin to screen for positive colonies. About 60% of those colonies were identified as positive using the colony PCR method. These results confirm that continuous genomic modifications can be successfully carried out using the UCM system without the step of curing selectable marker (Figure 3).

### Identification of the Carotenoid Production by Resulting Strains Subjected to Metabolic Engineering

The *gal* operon repressor, *Gal*R regulator, was deleted to increase the galactose content in the cell. Furthermore, glucose internalization by the phosphotransferase system (PTS) represents a drawback for the production of isoprene because the section of phosphoenol pyruvate was consumed during glucose phosphorylation in *E. coli*. Thus, the yield of carotenoids can be increased if *ptsG* gene is deleted. Besides, previous research demonstrated that *Glk* levels enabled a PTS^–^ strain to sustain high growth rates. Thus, the yield of isoprene would be increased in the cell with deleted *ptsG* but increased *Glk*. In addition, *pgi*-knockout strain demonstrated that the flux through the glyoxylate shunt was increased, while lycopene production was decreased in comparison with the parent strain. Double deletion of *galR* and *ptsG* genes can block PTS-mediated glucose uptake, leaving non-PTS glucose transport unaffected.

**FIGURE 4:**
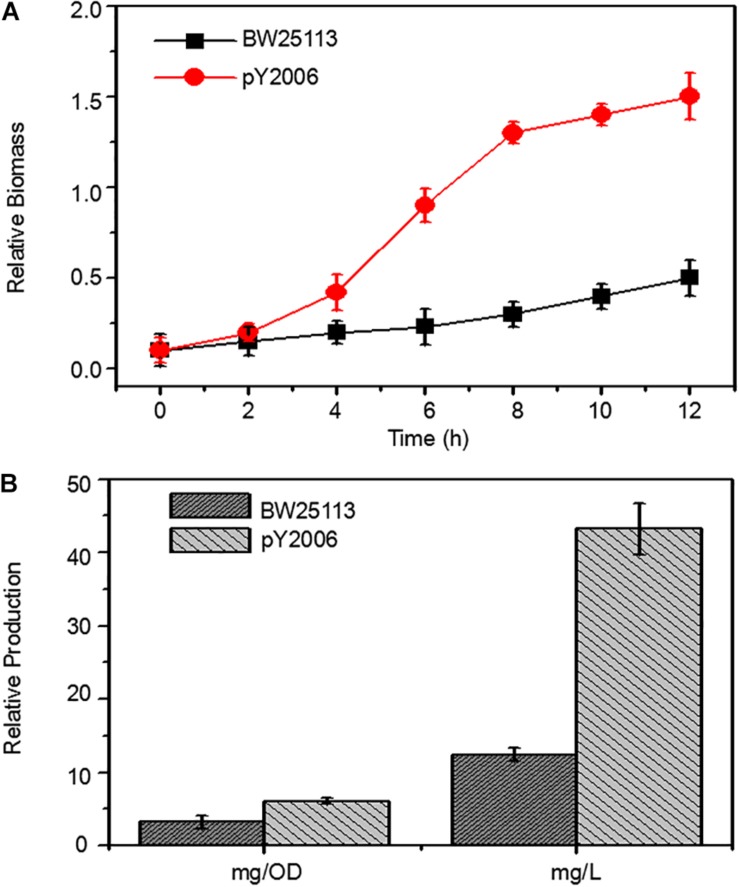
Metabolic engineering of *E. coli* to produce neurosporene. **(A)** The PY2006 cells (the genotypes shown in Table 1) with three knocked out genes grew faster than wild-type cell in Minimum Essential medium. **(B)** Comparison of neurosporene production in the engineered strains pY2006 and wild-type strain BW25113 in ZYM-5052 autoinducing medium. Data are expressed as the mean ± SD of three independent experiments.

To demonstrate the efficiency of the UCM method in synthetic biology, we developed a neurosporene-producing *E. coli* strain as a proof-of-concept example for the sequential deletion of multiple genes including *galR*, *ptsG*, and *pgi*. Meanwhile, the strain had increased content of *glk* genes. Neurosporene was produced by the triple-genes deleted strain. The cell growth curve shows that the final strain grew faster than the wild-type cells (Figure 4A). Notably, 43.24 g/L of neurosporene was produced by the final strain PY2006 (Figure 4B). It is emphasized that the sequential deletion of target genes took overall 10 days and that transformation and curing steps were not required. The antibiotic resistance gene was used repeatedly, which took about one-third of the time required during the application of the conventional methods.

## Discussion and Conclusion

This study describes the novel UAA mutagenesis system that utilizes a pair of exogenous *M. jannaschii tyrosyl-tRNA* and TyrRS construct. To confirm that the system runs effectively, three different experimental designs were used. First, to reduce the false-positive rate due to plasmid incompatibility, the vector pSBC1a-CtR and the template vector including pSLKM, pSLC, and pSLK2272 were constructed with low-copy number replication origin pSC101. When ampicillin and kanamycin were added to the plate, homologous recombination occurred. The cells lost the template vectors via the pressure of incompatibility during replication. The false-positive products of recombination were eliminated when antibiotic resistance gene was introduced as the selectable marker. Second, according to the previous study, once the expression of the TyRS was increased, overall yields of mutant proteins were also effectively increased (Hernández-Montalvo et al., 2003). C1 promoter (laboratory stocked) was used instead of glutamine promoter to increase the efficiency of the UAA mutagenesis system. The test data showed that TyrRS expression was the same as that of vector pBK under promoter *gln* and replication origin *ColE1* in cells where the TyrRS gene was present under the C1 constitute promoter (Young et al., 2010). Third, Glu160 site of aminoglycoside-3′-*O*-phosphotransferase gene is an enzyme activity site. 3-Iodo-tyrosine, which is slightly different from the normal amino acid, was introduced to Glu160 of aminoglycoside-3′-O-phosphotransferase gene using amber stop codon UAG. The mutated *lox*P sequences (*lox*LE/*lox*RE or *lox*2272) were used for the gene knockout studies (Usui et al., 2012) to avoid unwanted recombination events. In our study, three template plasmids, pSLKM, pSLC, and pSLK2272, were used for the generation of linear gene knockout DNA fragments in simultaneous triple-gene knockout experiments.

The new knockout system simplifies plasmid transformation using Cre recombinase induction and expression and Km^R^ gene elimination. When 3-iodotyrosine was added to the culture media, the mutational antibiotic Km^R^ gene was translated to the full-length protein, and the cells were able to grow in this medium. After the first selection stage, the mutants confirmed by colony PCR were restreaked on a plate containing mutation Km^R^ gene and pSBC1a-CtR at the second selection stage. Despite the gene knockout, DNA linear fragments with or without different antibiotic resistance gene were transferred into the cells. The plates without 3-iodotyrosine allowed the Km^R^ mutation gene in the chromosome to be translated to truncation, and *E. coli* cells did not grow due to the activity of antibiotic resistance genes. For the sequential multiple gene knockout, the Km^R^ genes were used as the selectable marker. Finally, cells were suspended in LB and were spread on a plate containing ampicillin and IPTG to induce *lox*P-mediated excision of the resistance marker. All colonies lost the resistance marker successfully. There were some limitations due to the limited number of resistance markers used for selection during simultaneous deletion approach. This problem could be lessened with the application of the developed gene knockout system with new plasmid pSBC1a-CtR. Gene deletions could be continued until the desired multiple-gene knockout mutants are obtained. The additional steps curing antibiotic resistance gene could be eliminated and the antibiotic label used many times without restriction of the antibiotic resistance gene.

The method based on the engineering of modular orthogonal UAA makes possible the genetic encoding of the UAAs in *E. coli.* Applications of genetically encoded UAAs are widespread. However, the applications were mostly used to address protein structure and function. This study introduces new tools to optimize the cell knockout/in system. Our procedure allows the sequential deletion of three genes in one week, a much shorter time period than previously required for the conventional *cre* recombinase mediated gene knockout method. In summary, using the UAA mutagenesis system, we developed a simple and highly efficient method of integrating DNA into chromosomal genes in *E. coli*. This method could be widely applied in gene manipulation.

## Data Availability Statement

The datasets generated for this study can be found in the https://www.ncbi.nlm.nih.gov/nuccore/NC_000913.3?report=genbank&from=4233758&to=4235407, https://www.ncbi.nlm.nih.gov/nuc core/NC_011750.1?report=genbank&from=3405133&to=3406164, https://www.ncbi.nlm.nih.gov/nuccore/NC_000913.3?report=genbank&from=1157869&to=1159302.

## Author Contributions

XX carried out the experiment and wrote the manuscript. HZ, WL, and YT analyzed the data and reviewed the manuscript.

## Conflict of Interest

HZ was employed by company Xiamen Huison Biotech Co., Ltd. The remaining authors declare that the research was conducted in the absence of any commercial or financial relationships that could be construed as a potential conflict of interest.

## Abbreviations

Ap^R^: ampicillin resistance gene
Cm^R^: chloramphenicol resistance gene
*E. coli*: *Escherichia coli;* IPTG isopropyl- β -D-thiogalactoside
Km^R^: kanamycin resistance gene
TyrRS: tyrosyl-tRNA synthetase
UAA: unnatural amino acid
UCM: UAA conditional selectable marker.
