# Supplementary material for: Extension of Genetic Marker List Using Unnatural Amino Acid System: An Efficient Genomic Modification Strategy in Escherichia coli
Source: Front Bioeng Biotechnol. 2020 Apr 28;8:145. doi: 10.3389/fbioe.2020.00145 (PMC7198746; doi:10.3389/fbioe.2020.00145)
Supplement: Supplementary file 1 [file Data_Sheet_1.pdf]

Extension of Genetic Marker List Using Unnatural Amino Acid System: an Efficient Genomic Modification Strategy in *Escherichia coli*

Xinyi Xu[1, 2], Huichang Zhong[3], Weifeng Liu[2*], and Yong Tao[2*]

[1] Engineering Research Center of Molecular and & Neuroimaging, Ministry of Education, School of Life Sciences and Technology, Xidian University, Xi'an, China,

[2] Chinese Academy of Sciences Key Laboratory of Microbial Physiological and Metabolic Engineering, Institute of Microbiology, Chinese Academy of Sciences, Beijing, China,

[3] Xiamen Huison Biotech Co., Ltd., Xiamen, China

[*]Correspondence:
Corresponding Author
Wei Feng Liu: liuwfv@163.com
Yong Tao: taoyong@im.ac.cn

Supplementary Data

Table S1. Oligonucleotides used in this study.

Name	Sequence (5' → 3')
Cre-KpnI-F	CATAGGTACCATGTCCAATTTACTGACC
Cre-EcoRI-R	CACTGAATTCCTAATCGCCATCTTCCAG
TyrRS-NcoI-F	CTACCATGGACGAATTTGAAATGATAAAG
TyrRS-XhoI-R	GTACTCGAGTTATAATCTCTTTCTAATTG
tRNA-1-F	GTCGAAAGACTGGGCCTTTCGTCGACATCCCCCATCAAAAAAAT
tRNA-1-R	GATGGGGGATGCGGCCGCAAAAAAAATCCTTAGCTTTC
tRNA-2-F	TTGCGGCCGCATCCCCCATCATCCCCCATC
tRNA-2-R	GATGGGGGATGGCGCGCCAAAAAAAATCCTTAGCTTTC
tRNA-3-F	TTGGCGCGCCATCCCCCATCATCCCCCATC
tRNA-3-R	GGGCGTTTTATGGCGGGTCTGGTCGACCGCCGGCGAAAAAAAT CCTTAGCTTTC
pKD-Cre-F	AAAACTGTCCATACCATGGACCTGCAGGGAGTCTGAGTCTGAGT CTCGCGCGCGGACACCATCGAATGGTGCAAAAC
pKD-Cre-R	AACAGACGAAGAATCCATGGCTAATCGCCATCTTCCAGCAG
C1-F	CTGTCCATACCATGGACCTGCAGGTTATCAAAAAGAGTATTGACA TA
tRNA-R	AGACTCAGACTCCCTGCAGGCACGGGCTTCTCAGGGCGTTT
At58-ispF-AscI-R	CATAGGCGCGCCTCATAGCGGTCTGCCTTGGTAGAC
At58-ispD-sbfI-F	CATACCTGCAGGAAGGAGATATACATGAAATTCGGCATCGTCATC
lox66Cm	GCAGCAAAACCCGCGGCCGCATAACTTCGTATAGCATACATTAT ACGAACGGTAAAGATCCCCTCACTCACTTCGCAGAATAAATAAA TC
lox71Cm	CTATACCAAGTTATGAAGCTAGCATAACTTCGTATAATGTATGCT ATACGAACGGTAAGAGCGCTTTTGAAGCTGCCTCAGGCATTTGA GAAGCACAC
pSLM-lox66-Kana	AAGCTTGAGCTCGCTAGCTTCATAACTTCGTATAGCATACATTAT ACGAACGGTAAAGATCCCCTCACGCTGCCG
pSLM-lox71-Kana	CCTGCAGACTAGTCTCGAGAATATCCTCCTTATAACTTCGTATAA TGTATGCTATACGAACGGTAAGAGCGCTTTTGAAGCTGGGGTG
lox2272Km-F	AAGCTTGAGCTCGCTAGCTTCATAACTTCGTATAAAGTATCCTAT ACGAAGTTATAAGATCCCCTCACGCTGCCG
lox2272Km-R	CCTGCAGACTAGTCTCGAGAATATCCTCCTTATAACTTCGTATAA AGTATCCTATACGAAGTTATAGAGCGCTTTTGAAGCTGGGGTG
Kam-F2	GACTAGGAGCATCAGGGGCTCGCGCC
Kam-R2	CTCCTAGTCCAGATCATCCTGATCGAC
kanaTAA-F	TTCTTGACGA GTTCTTCTAA
GalR- FC	CAACCTGAAGCCAAACGCCACCAGC
GalR-RC	ACCCGCGCCTTGCCGGAAGTGAAGG
PtsG-FC	CATATGTTTTGTCAAAATGTGCAAC

PtsG-RC	CCGCTACCAGGTGACGAAAAACTTC
Pgi-FC	CTCAACATTACGCTAACGGCAC
Pgi-RC	TTATCCTAAAACGCCATCGC
poxB-F1	TCAGATGAACTAAACTTGTTACCGTTATCACATTCAGGAGATGGA GAACCGCATGCCACAGCTAACACC
poxB-R1	CGTAAATCAATCATGGCATGTCCTTATTATGACGGGAAATGCCACC CTTTGACGGATCCCTGCAGACTA
GalR-F1	TTTTCCGTAACACTGAAAGAATGTAAGCGTTTACCCACTAAGGTA TTTTATTCCGGGGATCCGTCGACC
GalR-R1	GTCGCCAGACCATCGAAGAATTACTGGCGCTGGAATTGCTTTAAC TGCGGATGTAGGCTGGAGCTGCTTCG
ptsG-F1	CCCCCCTTGCCACGCGTGAGAACGTAAAAAAAGCACCCATACTC AGGAGCACTCTCAATTGCATGCCACAGCTAACACC
ptsG-R1	AGCCATCTGGCTGCCTTAGTCTCCCCAACGTCTTACGGATTAGTG GTTACGGATGTACTCGACGGATCCCTGCAGACTA
pgi-F1	TACAATCTTCCAAAGTCACAATTCTCAAAATCAGAAGAGTATTGC TAATGATTCCGGGGATCCGTCGACC
pgi-R1	GCCTTATCCGGCCTACATATCGACGATGATTAACCGCGCCACGCTT TATATGTAGGCTGGAGCTGCTTCG
p119F	TTGAATTCGGTACCAAGCTTGCATGCCACAGCTAACACCACG
TrrnB-R	CAGTCGAAAGACTGGGCCTTGCTAGCTTCATAACTTGGTATAG

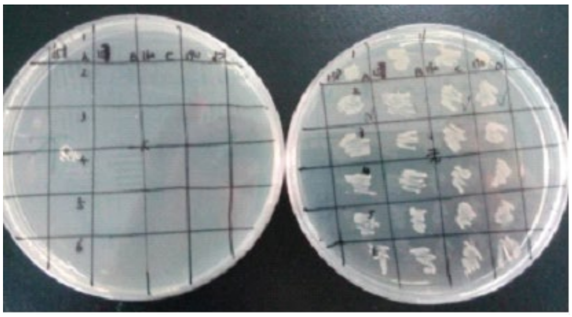

Figure S1. The BW25113 cells harboring pC1RT vector and either of the pSLM plasmids that contained mutated APH genes. Left: The plate contained Km. Right: The plate contained Km and 3-iodo-L-tyrosine (1mM).

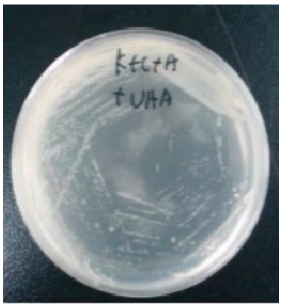

Figure S2. The BW25113 cells contanied three plasmids including pLSM with APH 160 site mutation, pC1, pKD46 coated on plates containing Km, Cm, Ap, and 3-iodotyrosine.

Table S2. The sequence of cloning of plasmid pSLC and pSLK2272.

MCS1-Lox66:	GCGGCCGCGGTTGAATTCGGTACCAAGCTTGAGCTCGCTAGCTTCATAACTTGGTATAGCATACATTATACGAACGGTA
Lox71-MCS2:	TACCGTTCGTATAGCATACATTATACGAAGTTATAAGGAGGATATTCTCGAGACTAGTCTGCAGGGATCCGTCGACGGTTGGCGCGCC
MCS1-Lox2272:	GCGGCCGCGGTTGAATTCGGTACCAAGCTTGAGCTCGCTAGCTTCATAACTTCGTATAAAGTATCCTATACGAAGTTA
Lox2272-MCS2:	ATAACTTCGTATAAAGTATCCTATACGAAGTTATAAGGAGGATATTCTCGAGACTAGTCTGCAGGGATCCGTCGACGGTTGGCGCGCC

Table S3. The sequence of plasmid pSBC1A-CtR.

accacttcggattatcccgtgacaggtcattcagactggctaatgcacccagtaaggcagcggtatcatcaacggggtctgacgctcagtgga
acgaaaactcacgttaagggattttggtcatgagattatcaaaaaggatcttcacctagatccttttaaattaaaaatgaagttttaaatcaatctaa
agtatatattgagtaaacttggtctgacagttaccaatgcttaatcagtgaggcacctatctcagcgatctgtctatttcgttcatccatagttgcctg
actccccgtcgtgtagataactacgatacgggagggcttaccatctggccccagtgctgcaatgataccgcgagacccacgctcaccggctc
cagatttatcagcaataaaccagccagccggaagggccgagcgcagaagtggtcctgcaactttatccgcctccatccagtctattaattgttg
ccgggaagctagagtaagtagttcgccagttaatagtttgcgcaacgttgttgccattgctacaggcatcgtggtgtcacgctcgtcgtttggtat
ggcttcattcagctccggttcccaacgatcaaggcgagttacatgatcccccatgttgtgcaaaaaagcggttagctccttcggtcctccgatcg
ttgtcagaagtaagttggccgcagtgttatcactcatggttatggcagcactgcataattctcttactgtcatgccatccgtaagatgcttttctgtga
ctggtgagtactcaaccaagtcattctgagaatagtgtatgcggcgaccgagttgctcttgcccggcgtcaatacgggataataccgcgccac
atagcagaactttaaaagtgctcatcattggaaaacgttcttcggggcgaaaactctcaaggatcttaccgctgttgagatccagttcgatgtaac
ccactcgtgcacccaactgatcttcagcatcttttactttcaccagcgtttctgggtgagcaaaaacaggaaggcaaaatgccgcaaaaaaggg
aataagggcgacacggaaatgttgaatactcatactcttcctttttcaatattattgaagcatttatcagggttattgtctcatgagcggatacatattt
gaatgtatttagaaaaataaacaaataggggttccgcgcacatttccccgaaaagtgccacctgttatgacaacttgacggctacatcattcactt
tttcttcacaaccggcacggaactcgctcgggctggccccggtgcatttttaaatacccgcgagaaatagagttgatcgtcaaaaccaacattg
cgaccgacggtggcgataggcatccgggtggtgctcaaaagcagcttcgcctggctgatacgttggtcctcgcgccagcttaagacgctaat
ccctaactgctggcggaaaagatgtgacagacgcgacggcgacaagcaaacatgctgtgcgacgctggcgatatcaaaattgctgtctgcc
aggtgatcgctgatgtactgacaagcctcgcgtacccgattatccatcggtggatggagcgactcgttaatcgcttccatgcgccgcagtaaca
attgctcaagcagatttatcgccagcagctccgaatagcgcccttccccttgcccggcgttaatgatttgcccaaacaggtcgctgaaatgcgg
ctggtgcgcttcatccgggcgaaagaaccccgtattggcaaatattgacggccagttaagccattcatgccagtaggcgcgcggacgaaagt
aaacccactggtgataccattcgcgagcctccggatgacgaccgtagtgatgaatctctcctggcgggaacagcaaaatatcacccggtcgg
caaacaaattctcgtccctgatttttcaccaccccctgaccgcgaatggtgagattgagaatataacctttcattcccagcggtcggtcgataaaa
aaatcgagataaccgttggcctcaatcggcgttaaacccgccaccagatgggcattaaacgagtatcccggcagcaggggatcattttgcgct
tcagccatacttttcatactcccgccattcagagaagaaaccaattgtccatattgcatcagacattgccgtcactgcgtcttttactggctcttctc
gctaaccaaaccggtaaccccgcttattaaaagcattctgtaacaaagcgggaccaaagccatgacaaaaacgcgtaacaaaagtgtctataa
tcacggcagaaaagtccacattgattatttgcacggcgtcacactttgctatgccatagcatttttatccataagattagcggatcctacctgacgc
tttttatcgcaactctctactgtttctccatacccgtttttttgggaattcgagctctaaggaggttataaaaaatggatattaatactgaaactgagat
caagcaaaagcattcactaacccccctttcctgttttcctaatcagcccggcatttcgcgggcgatattttcacagctatttcaggagttcagccatg
aacgcttattacattcaggatcgtcttgaggctcagagctgggcgcgtcactaccagcagctcgcccgtgaagagaaagaggcagaactggc
agacgacatggaaaaaggcctgccccagcacctgtttgaatcgctatgcatcgatcatttgcaacgccacggggccagcaaaaaatccatta
cccgtgcgtttgatgacgatgttgagtttcaggagcgcatggcagaacacatccggtacatggttgaaaccattgctcaccaccaggttgatatt
gattcagaggtataaaacgaatgagtactgcactcgcaacgctggctgggaagctggctgaacgtgtcggcatggattctgtcgacccacag
gaactgatcaccactcttcgccagacggcatttaaaggtgatgccagcgatgcgcagttcatcgcattactgatcgttgccaaccagtacggcc

ttaatccgtggacgaaagaaatttacgcctttcctgataagcagaatggcatcgttccggtggtgggcgttgatggctggtcccgcatcatcaat
gaaaaccagcagtttgatggcatggactttgagcaggacaatgaatcctgtacatgccggatttaccgcaaggaccgtaatcatccgatctgc
gttaccgaatggatggatgaatgccgccgcgaaccattcaaaactcgcgaaggcagagaaatcacggggccgtggcagtcgcatcccaaa
cggatgttacgtcataaagccatgattcagtgtgcccgtctggccttcggatttgctggtatctatgacaaggatgaagccgagcgcattgtcga
aaatactgcatacactgcagaacgtcagccggaacgcgacatcactccggttaacgatgaaaccatgcaggagattaacactctgctgatcg
ccctggataaaacatgggatgacgacttattgccgctctgttcccagatatttcgccgcgacattcgtgcatcgtcagaactgacacaggccga
agcagtaaaagctcttggattcctgaaacagaaagccgcagagcagaaggtggcagcatgacaccggacattatcctgcagcgtaccggga
tcgatgtgagagctgtcgaacaggggggatgatgcgtggcacaaattacggctcggcgtcatcaccgcttcagaagttcacaacgtgatagca
aaaccccgctccggaaagaagtggcctgacatgaaaatgtcctacttccacaccctgcttgctgaggtttgcaccggtgtggctccggaagtt
aacgctaaagcactggcctggggaaaacagtacgagaacgacgccagaaccctgtttgaattcacttccggcgtgaatgttactgaatcccc
gatcatctatcgcgacgaaagtatgcgtaccgcctgctctcccgatggtttatgcagtgacggcaacggccttgaactgaaatgcccgtttacct
cccgggatttcatgaagttccggctcggtggtttcgaggccataaagtcagcttacatggcccaggtgcagtacagcatgtgggtgacgcgaa
aaaatgcctggtactttgccaactatgacccgcgtatgaagcgtgaaggcctgcattatgtcgtgattgagcgggatgaaaagtacatggcga
gttttgacgagatcgtgccggagttcatcgaaaaaatggacgaggcactggctgaaattggttttgtatttggggagcaatggcgatgatgattg
agcgggatgaaaagtacatggcgagttttgacgagatcgtgccggagttcatcgaaaaaatggacgaggcactggctgaaattggttttgtatt
tggggagcaatggcgatgacgcatctgattgagcgggatgaaaagtacatggcgagttttgacgagatcgtgccggagttcatcgaaaaaat
ggacgaggcactggctgaaattggttttgtatttggggagcaatggcgatgacgcatcacgcatcctcacgataatatccgggtaggcgcaat
cactttcgtctactccgttacaaagcgaggctgggtatttcccggcctttctgttatccgaaatccactgaaagcacagcggctggctgaggaga
taaataataaacgaggggctgtatgcacaaagcatcttctgttgagttaagaacgagtatcgagatggcacatagccttgctcaaattggaatca
ggtttgtgccaataccagtagaaacagacgaagaatgcacagcggctggctgaggagataaataataaacgaggggctgtatgcacaaagc
atcttctgttgagttaagaacgagtatcgagatggcacatagccttgctcaaattggaatcaggtttgtgccaataccagtagaaacagacacgc
acagcggctggctgaggagataaataataaacgaggggctgtatgcacaaagcatcttctgttgagttaagaacgagtatcgagatggcacat
agccttgctcaaattggaatcaggtttgtgccaataccagtagaaacagacacgcaaatgctgaatgagggcatcgttcccactgcgatgctg
gttgccaacgatcagatggcgctgggcgcaatgcgcgccattaccgagtccgggctgcgcgttggtgcggatatctcggtagtgggatacg
acgataccgaagacagctcatgttatatcccgccgtcaaccaccatcaaacaggattttcgcctgctggggcaaaccagcgtggaccgcttgc
tgcaactctctcagggccaggcggtgaagggcaatcagctgttgcccgtctcactggtgaaaagaaaaaccaccctggcgcccaatacgca
aaccgcctctccccgcgcgttggccgattcattaatgcagctggcacgacaggtttcccgactggaaagcgggcagtgagcgcaacgcaatt
aatgtgagttagctcactcattaggcaccccaggctttacactttatgcttccggctcgtatgttgtgtggaattgtgagcggataacaatttcaca
caggaaacagctatgaccatgattacggattcactggccgtcgttttacaacgtcgtgactgggaaaaccctggcgttacccaacttaatcgcct
tgcagcacatcccccttcgccagctggcgtaatagcgaagaggcccgcaccgatcgcccttcccaacagttgcgcagcctgaatggcgaat
ggcgctttgcctggtttccggcaccagaagcggtgccggaaagctggctggagtgcgatcttcctgaggccgatactgtcgtcgtcccctcaa
actggcagatgcacggttacgatgcgcccatctacaccaacgtaacctatcccattacggtcaatccgccgtttgttcccacggagaatccgac
gggttgttactcgctcacatttaatgttgatgaaagctggctacaggaaggccagacgcgaattatttttgatggcgttggaattacgttatcgact
gcacggtgcaccaatgcttctggcgtcaggcagccatcggaagctgtggtatggctgtgcaggtcgtaaatcactgcataattcgtgtcgctca
aggcgcactcccgttctggataatgttttttgcgccgacatcataacggttctggcaaatattctgaaatgagctgttgacaattaatcatcggctc
gtataatgtgtggaattgtgagcggataacaatttcacacaggaaacagtattccatgaaaactgtccataccatggacctgcagggagtctga
gtctgagtctcgcgcgcggacaccatcgaatggtgcaaaacggtaccatgtccaatttactgaccgtacaccaaaatttgcctgcattaccggt
cgatgcaacgagtgatgaggttccaagaacctgatggacatgttcagggatcgccaggcgttttctgagcatacctggaaaatgcttctgtccg
tttgccggtcgtgggcggcatggtgcaagttgaataaccggaaatggtttcccgcagacctgaagatgttcgcgattatcttctatatcttcaggc
gcgcggtctggcagtaaaaactatccagcaacatttgggccagctaaacatgcttcatcgtcggtccgggctgccacgaccaagtgacagca
atgctgtttcactggttatgcggcggatccgaaaagaaaacgttgatgccggtgaacgtgcaaaacaggctctagcgttcgaacgcactgattt
cgaccaggttcgttcctcatggaaaatagcgatcgctgccaggatatacgtaatctggcatttctggggattgcttataacaccctgttacgtata
gccgaaattgccaggatcagggttaaagatatctcacgtactgacggtgggagaatgttaatccatattggcagaacgaaaacgctggttagc
accgcggtgtagagaaggcacttagcctgggggtaactaaactggtcgagcgatggatttccgtctctggtgtagctgatgatccgaataacta

cctgttttgccgggtcagaaaaaatggtgttgccgcgccatctgccaccagccagctatcaactcgcgccctggaagggatttttgaagcaact
catcgattgatttacggcgctaaggatgactctggtcagagatacctggcctggtctggacacagtgcccgtgtcggagccgcgcgagatatg
gcccgcgctggagtttcaataccggagatcatgcaagctggtggctggaccaatgtaaatattgtcatgaactatatccgtaacctggatagtg
aaacaggggcaatggtgcgcctgctggaagatggcgattaggaattcctgctggaagatggcgattagccatggattcttcgtctgttatgttaa
tccatattggcagaacgaaaacgctggttagcaccgcaggtgtagagaaggcacttagcctgggggtaactaaactggtcgagcgatggatt
tccgtctctggtgtagctgatgatccgaataactacctgttttgccgggtcagaaaaaatggtgttgccgcgccatctgccaccagccagctatc
aactcgcgccctggaagggatttttgaagcaactcatcgattgaagcaactcatcgatttgatttacggcgctaaggatgactctggtcagagat
acctggcctggtctggacacagtgcccgtgtcggagccgcgcgagatatggcccgcgctggagtttcaataccggagatcatgcaagctgg
tggctggaccaatgtaaccatggacgaatttgaaatgataaagagaaacacatctgaaattatcagcgaggaagagttaagagaggttttaaaa
aaagatgaaaaatctgctatgataggttttgaaccaagtggtaaaatacatttagggcattatctccaaataaaaaagatgattgatttacaaaatg
ctggatttgatataattataccgttggctgatttagctgcctatttaaaccagaaaggagagttggatgagattagaaaaataggagattataacaa
aaaagtttttgaagcaatggggttaaaggcaaaatatgtttatggaagtgaagagccgcttgataaggattatacactgaatgtctatagattggc
tttaaaaactaccttaaaaagagcaagaaggagtatggaacttatagcaagagaggatgaaaatccaaaggttgctgaagttatctatccaata
atgcaggttaatagtactcattatcatggcgttgatgttgcagttggagggatggagcagagaaaaatacacatgttagcaagggagcttttacc
aaaaaaggttgtttgtattcacaaccctgtcttaacgggtttggatggagaaggaaagatgagttcttcaaaagggaattttatagctgttgatgac
tctccagaagagattagggctaagataaagaaagcatactgcccagctggagttgttgaaggaaatccaataatggagatagctaaatacttcc
ttgaatatcctttaaccataaaaaggccagaaaaatttggtggagatttgacagttaatagctatgaggagttagagagtttatttaaaaataagga
attgcatccaatggatttaaaaaatgctgtagctgaagaacttataaagattttagagccaattagaaagagattataactcgagggtagatctgg
tactagtggtgaattcggtgagctcggtctgcagctggtgccgcgcggcagccaccaccaccaccaccactaatacagattaaatcagaacg
cagaagcggtctgataaaacagaatttgcctggcggcagtagcgcggtggtcccacctgaccccatgccgaactcagaagtgaaacgccgt
agcgccgatggtagtgtggggtctccccatgcgagagtagggaactgccaggcatcaaataaaacgaaaggctcagtcgaaagactgggc
ctttcgtcgacatcccccatcaaaaaaatattctcaacataaaaaactttgtgtaatacttgtaacgctgaattcccggcggtagttcagcagggc
agaacggcggactctaaatccgcatggcgctggttcaaatccggcccgccggaccactgcagatccttagcgaaagctaaggattttttttgc
ggccgcatcccccatcaaaaaaatattctcaacataaaaaactttgtgtaatacttgtaacgctgaattcccggcggtagttcagcagggcagaa
cggcggactctaaatccgcatggcgctggttcaaatccggcccgccggaccactgcagatccttagcgaaagctaaggattttttttggcgcg
ccatcccccatcaaaaaaatattctcaacataaaaaactttgtgtaatacttgtaacgctgaattcccggcggtagttcagcagggcagaacggc
ggactctaaatccgcatggcgctggttcaaatccggcccgccggaccactgcagatccttagcgaaagctaaggattttttttcgccggcggtc
gaccagacccgccataaaacgccctgaacagttgttctacttttgtttgttagtcttgatgcttcactgatagatacaagagccataagaacctca
gatccttccgtatttagccagtatgttctctagtgtggttcgttgtttttgcgtgagccatgagaacgaaccattgagatcatacttactttgcatgtca
ctcaaaaattttgcctcaaaactggtgagctgaatttttgcagttaaagcatcgtgtagtgtttttcttagtccgttacgtaggtaggaatctgatgta
atggttgttggtattttgtcaccattcatttttatctggttgttctcaagttcggttacgagatccatttgtctatctagttcaacttggaaaatcaacgtat
cagtcgggcggcctcgcttatcaaccaccaatttcatattgctgtaagtgtttaaatctttacttattggtttcaaaacccattggttaagccttttaaa
ctcatggtagttattttcaagcattaacatgaacttaaattcatcaaggctaatctctatatttgccttgtgagttttcttttgtgttagttcttttaataacc
actcataaatcctcatagagtatttgttttcaaaagacttaacatgttccagattatattttatgaattttttaactggaaaagataaggcaatatctctt
cactaaaaactaattctaatttttcgcttgagaacttggcatagtttgtccactggaaaatctcaaaagcctttaaccaaaggattcctgatttccaca
gttctcgtcatcagctctctggttgctttagctaatacaccataagcattttccctactgatgttcatcatctgagcgtattggttataagtgaacgata
ccgtccgttctttccttgtagggttttcaatcgtggggttgagtagtgccacacagcataaaattagcttggtttcatgctccgttaagtcatagcga
ctaatcgctagttcatttgctttgaaaacaactaattcagacatacatctcaattggtctaggtgattttaatcactataccaattgagatgggctagt
caatgataattactagtccttttcctttgagttgtgggtatctgtaaattctgctagacctttgctggaaaacttgtaaattctgctagaccctctgtaaa
ttccgctagacctttgtgtgttttttttgtttatattcaagtggttataatttatagaataaagaaagaataaaaaaagataaaaagaatagatcccagc
cctgtgtataactcactactttagtcagttccgcagtattacaaaaggatgtcgcaaacgctgtttgctcctctacaaaacagaccttaaaaccctа
aaggcttaagtagcaccctcgcaagctcggttgcggccgcaatcgggcaaatcgctgaatattccttttgtctccgaccatcaggcacctgagt
cgctgtctttttcgtgacattcagttcgctgcgctcacggctctggcagtgaatgggggtaaatggcactacaggcgccttttatggattcatgca
aggaaactacccataatacaagaaaagcccgtcacgggcttctcagggcgttttatggcgggtctgctatgtggtgctatctgacttttgctgtt

cagcagttcctgccctctgattttccagtct
